# Fusion of Lysostaphin to an Albumin Binding Domain Prolongs Its Half-Life and Bactericidal Activity in the Systemic Circulation

**DOI:** 10.3390/molecules24162892

**Published:** 2019-08-09

**Authors:** Alexander V. Grishin, Nikita V. Shestak, Natalia V. Lavrova, Alexander M. Lyashchuk, Liubov I. Popova, Natalia V. Strukova, Maria S. Generalova, Anna V. Ryazanova, Nikita B. Polyakov, Zoya M. Galushkina, Lyubov A. Soboleva, Irina S. Boksha, Anna S. Karyagina, Vladimir G. Lunin

**Affiliations:** 1 N. F. Gamaleya National Research Center of Epidemiology and Microbiology, Ministry of Health of the Russian Federation, 123098 Moscow, Russia; 2 All-Russia Research Institute of Agricultural Biotechnology, 127550 Moscow, Russia; 3 MIREA Russian Technological University, 119048 Moscow, Russia; 4 Vernadsky Institute of Geochemistry and Analytical Chemistry, Russian Academy of Sciences, 119991 Moscow, Russia; 5 Mental Health Research Center, 115522 Moscow, Russia; 6 A. N. Belozersky Institute of Physico-Chemical Biology, M.V. Lomonosov Moscow State University, 119992 Moscow, Russia

**Keywords:** antibiotic resistance, endolysin, lysin, lysostaphin, albumin-binding domain, pharmacokinetics, pharmacodynamics

## Abstract

Antibacterial lysins are promising proteins that are active against both antibiotic-susceptible and antibiotic-resistant bacterial strains. However, a major limitation of antibacterial lysins is their fast elimination from systemic circulation. PEGylation increases the plasma half-life of lysins but renders them inactive. Here we report the construction of a fusion protein of lysostaphin, a potent anti-staphylococcal lysin, and an albumin-binding domain from streptococcal protein G. The resulting fusion protein was less active than the parent enzyme lysostaphin, but it still retained significant antibacterial activity even when bound to serum albumin. The terminal half-life of the fusion protein in rats was five-fold greater than that of lysostaphin (7.4 vs. 1.5 h), and the area under the curve increased more than 115 times. Most importantly, this increase in systemic circulation time compensated for the decrease in activity. The plasma from rats that received an injection of the fusion protein retained bactericidal activity for up to 7 h, while plasma from rats that received plain lysostaphin lacked any detectable activity after 4 h. To the best of our knowledge, this is the first report of an antibacterial lysin with both improved pharmacokinetic parameters and prolonged bactericidal activity in the systemic circulation.

## 1. Introduction

Pharmaceutical companies cannot keep up with the continuous emergence and spread of resistant bacterial strains and provide novel antibiotics to the market, and alternative therapeutic strategies need to be explored [1]. Antibacterial lysins are considered a prospective class of compounds that are active against both antibiotic-resistant and antibiotic-susceptible strains of bacterial pathogens, and many natural and engineered lysins with different specificities have been described [2,3,4]. These enzymes degrade the peptidoglycan of the bacterial cell wall, which leads to bacterial lysis and death. The efficacy of both native and engineered lysins was demonstrated in numerous studies in experimental animal models of infections, including staphylococcal and streptococcal bacteremia, endocarditis and pneumonia, staphylococcal osteomyelitis, mastitis, burn wounds infections, and several others [4,5]. Two anti-staphylococcal lysins, CF-301 and SAL-1, have been tested in clinical trials with doses up to 0.4 and 10 mg/kg, respectively, administered by intravenous infusion and were found to be well tolerated [6,7]. The antibacterial lysins typically demonstrate rapid bactericidal activity, high specificity, harmlessness towards commensal flora, and synergy with antibiotics and antimicrobial peptides [2,8,9]. Unlike small molecule antibiotics, lysins can eradicate bacterial biofilms [10]. Moreover, lysins typically demonstrate a low probability of resistance development in vitro, and it is generally thought that resistant strains will rarely emerge in vivo as well [3,11].

Lysostaphin is a glycyl-glycine endopeptidase that cleaves the pentaglycine cross-bridges in staphylococcal peptidoglycan [12] and is one of the most potent antibacterial lysins described to date. Lysostaphin is a two-domain protein with an N-terminal catalytic domain that hydrolyzes the peptidoglycan and C-terminal peptidoglycan-binding domain that targets the enzyme to the cell wall. A minimum inhibitory concentration (MIC) of lysostaphin as low as 0.002 µg/mL was reported for several *Staphylococcus aureus* strains, with MIC_90_ of 0.031 µg/mL [13]. The relevance of *S. aureus* as a pathogen is largely defined by the existence of methicillin-resistant (MRSA) strains [14]. Importantly, lysostaphin kills both methicillin-sensitive and methicillin-resistant strains alike. Lysostaphin was shown to be effective in treating systemic staphylococcal infections in adult and neonatal mice and rats [15,16,17], eradicating bacterial biofilms from catheters [18] and preventing catheter colonization [19], reducing the mortality in mice with MRSA pneumonia [20], curing MRSA keratitis and decreasing bacterial counts in experimental endophthalmitis model in rabbits [21,22], and clearing colonizing *S. aureus* from the cotton rat nares [23]. Altogether, this makes lysostaphin a highly promising anti-staphylococcal molecule. However, after intravenous injection, lysostaphin is quickly eliminated from systemic circulation. The serum half-life of lysostaphin was estimated to be less than one hour in mice [24], and the terminal half-life of lysostaphin in rats was 1.5 h [25]. This necessitates administration of high doses and limits its systemic application.

Currently, there are several widely used approaches to increase the serum half-life of therapeutic proteins. The most frequently used methodology is the covalent conjugation of one or more polyethylene glycol chains (PEGylation). PEGylation protects the protein from proteolysis, reduces its immunogenicity, and increases its hydrodynamic radius. The latter leads to a less efficient glomerular filtration and longer residence time in the systemic circulation [26]. Unfortunately, PEGylation often negatively affects the activity of the modified protein. This effect was demonstrated for lysostaphin [24]. Conjugation of two or more 40 kDa PEG chains led to a dramatic decrease in lysostaphin staphylolytic activity. A similar effect was shown for another antibacterial lysin Cpl-1 [27].

Another increasingly used approach in extending the plasma half-life is the genetic fusion of a protein to the serum albumin [28]. Albumin is a large protein with a long in vivo half-life, and conjugation to albumin increases the effective hydrodynamic radius of a protein similarly to PEGylation. Moreover, when albumin is engulfed in lysosomes, it is recycled due to the interaction with FcRn receptors, and albumin-bound proteins can be recycled as well [29]. An albumin-fusion variant of glucagon-like peptide 1 has been approved for clinical use, and several other albumin-fusion therapeutic proteins are in development [28]. Genetic fusion to a small albumin-binding peptide or domain is an alternative to genetic fusion to albumin [29]. Several albumin-binding peptides and domains have been described, such as the albumin-binding domain from streptococcal protein G [29], engineered ankyrin repeat protein [30], short peptides [31,32], and single-domain antibodies [33,34,35]. Unlike PEGylation, which requires specific conditions to control the placement of the PEG conjugation and the amount of attached PEG chains, albumin binding allows easy control of both parameters by placing the albumin-binding domain at the desired position within the protein sequence. Interestingly, it was reported that binding to albumin decreases protein immunogenicity [36].

In this work, we attempted to extend the serum half-life of lysostaphin by fusing it to an albumin-binding domain of streptococcal protein G [37]. In the absence of albumin, the resulting modified lysostaphin was only 1.5 times less active than the parent enzyme, and it still retained 25% of the activity when bound to serum albumin. The residence time in the systemic circulation of this protein was dramatically increased compared to lysostaphin. Importantly, the improved pharmacokinetics resulted in improved ex vivo pharmacodynamics and prolonged bactericidal activity of the plasma samples, despite the decreased staphylolytic activity of the fusion protein. To the best of our knowledge, this is the first report of a modified antibacterial lysin with prolonged bactericidal activity in the systemic circulation.

## 2. Results

### 2.1. Lst-ABD Construction and Purification

**Figure 1:**
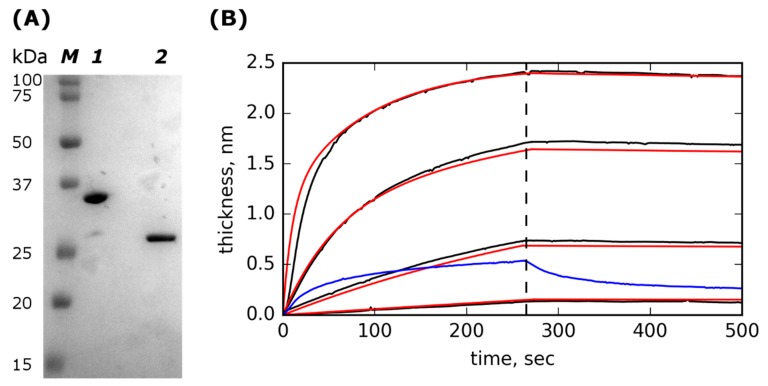
SDS-PAGE of purified Lst-ABD and kinetics of rat serum albumin (RSA) interactions with the immobilized Lst-ABD. (**A**) SDS-PAGE of purified Lst-ABD (lane 1) and lysostaphin (lane 2); lane M contains molecular mass standards with their molecular masses indicated on the left. (**B**) Kinetics of rat serum albumin (RSA) interactions with the immobilized Lst-ABD. Experimental curves corresponding to different concentrations of RSA in the solution are shown in black, fitted theoretical curves are shown in red; concentrations of RSA are (top to bottom) 250, 42, 7, and 1.2 µg/mL. The dashed vertical line marks the end of the association phase and the start of the dissociation phase. The binding and dissociation of 1 mg/mL bovine serum albumin (BSA) are shown in blue.

We constructed a recombinant fusion protein with lysostaphin at the N-terminus and the albumin-binding domain at the C-terminus [37]. This protein is further referred to as Lst-ABD. The albumin-binding domain from *Streptococcus* sp. protein G is 46 amino acids long three-helix bundle domain that binds to rat, human, and murine albumins with high affinity, and it shows weak or no interaction with bovine albumin [38,39]. The recombinant protein was produced in *Escherichia coli* and purified by cation-exchange chromatography. The Lst-ABD protein was of >95% purity with minute amounts of unmodified lysostaphin, probably resulting from degradation of the protein during synthesis or purification (Figure 1A). The correspondence of the purified protein to the theoretical sequence of Lst-ABD was confirmed by mass spectrometry analysis.

### 2.2. Lst-ABD Binds Rat Serum Albumin with Nanomolar Affinity

Next, we tested the ability of Lst-ABD to bind albumin. Kinetics of association and dissociation of rat serum albumin (RSA) and immobilized Lst-ABD were recorded using a photonic crystal-based biosensor [40] (Figure 1B). The obtained kinetic curves were well described by a two-site heterogenous binding model with identical *k_off_* values for both sites and 10-fold different *k_on_* values (Table 1). The high-affinity site had a *K*_D_ of 2.3 ± 0.95 nM, indicating strong binding of Lst-ABD to RSA. Bovine serum albumin demonstrated very weak binding and quick dissociation even at a very high concentration of 1 mg/mL (Figure 1B). The inverted orientation with immobilized RSA was not possible since both lysostaphin and Lst-ABD interacted nonspecifically with the carboxylate-derivatized chip surface due to their high positive charges.

### 2.3. Lst-ABD Kills Staphylococci Both in the Absence and in the Presence of Albumin

We further measured the antibacterial activity of Lst-ABD and lysostaphin on 7 methicillin-sensitive and 5 methicillin-resistant *S. aureus* strains. The minimum inhibitory concentrations (MICs) of Lst-ABD, which is the minimum concentration of an antibiotic that suppresses bacterial growth in a rich medium, were 8–64 times higher than the MICs of lysostaphin (Table 2). Interestingly, the addition of 50 µg/mL RSA increased the MICs of Lst-ABD by no more than one two-fold dilution. Minimal bactericidal concentrations (MBCs) of lysostaphin and Lst-ABD were equal to the corresponding MICs in most cases. Both MSSA and MRSA strains were killed by Lst-ABD with similar efficacy. One MSSA strain (Z 715-18) had an Lst-ABD MIC greater than the maximal tested concentration, and one MRSA strain (247G) had a high MIC of 12.8 µg/mL. Both strains were also less susceptible to lysostaphin.

**Figure 2:**
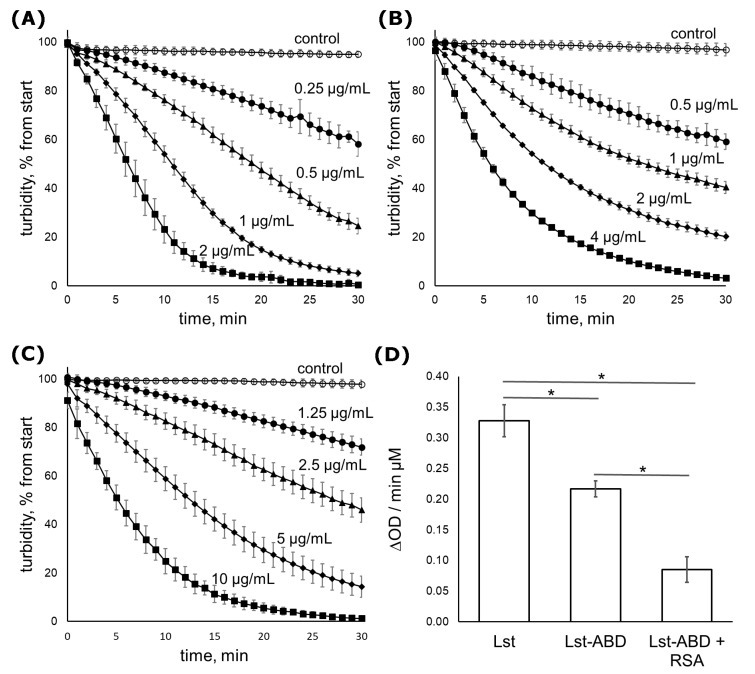
Time-dependent clearing of *S. aureus* ATCC 29,213 cell suspension by lysostaphin and Lst-ABD. (**A**) Lysostaphin: 2 µg/mL (squares), 1 µg/mL (diamonds), 0.5 µg/mL (triangles), 0.25 µg/mL (circles), or control (no protein, open circles). (**B**) Lst-ABD: 4 µg/mL (squares), 2 µg/mL (diamonds), 1 µg/mL (triangles), 0.5 µg/mL (circles), or control (no protein, open circles). (**C**) Lst-ABD in the presence of RSA: 10 µg/mL (squares), 5 µg/mL (diamonds), 2.5 µg/mL (triangles), 1.25 µg/mL (circles), or control (no protein, open circles). The protein concentrations are also indicated in the figure above the corresponding curves (**D**) The rate of cell suspension clearing adjusted to 1 µM of protein. Mean values from three independent experiments are shown, error bars represent standard deviation. Statistical significance was assessed by one-way ANOVA with Tukey’s Honest Significant Difference post-hoc analysis. All differences between the means were significant with *p* < 0.01. Lysostaphin data are reproduced from [25].

The kinetics of *S. aureus* cell lysis by Lst-ABD was followed in a turbidimetry assay using *S. aureus* strain ATCC 29213. In this assay, clearing of the bacterial cell suspension due to cell lysis by an antibacterial lysin is followed over time by monitoring the optical density at 550 nm (Figure 2A–C). In the absence of rat serum albumin, Lst-ABD cleared the cell suspension 1.5 times slower than lysostaphin. When bound to albumin, the activity of Lst-ABD was further decreased to about 25% of the lysostaphin activity (Figure 2D).

### 2.4. Lst-ABD Has an Improved Pharmacokinetic Profile

**Figure 3:**
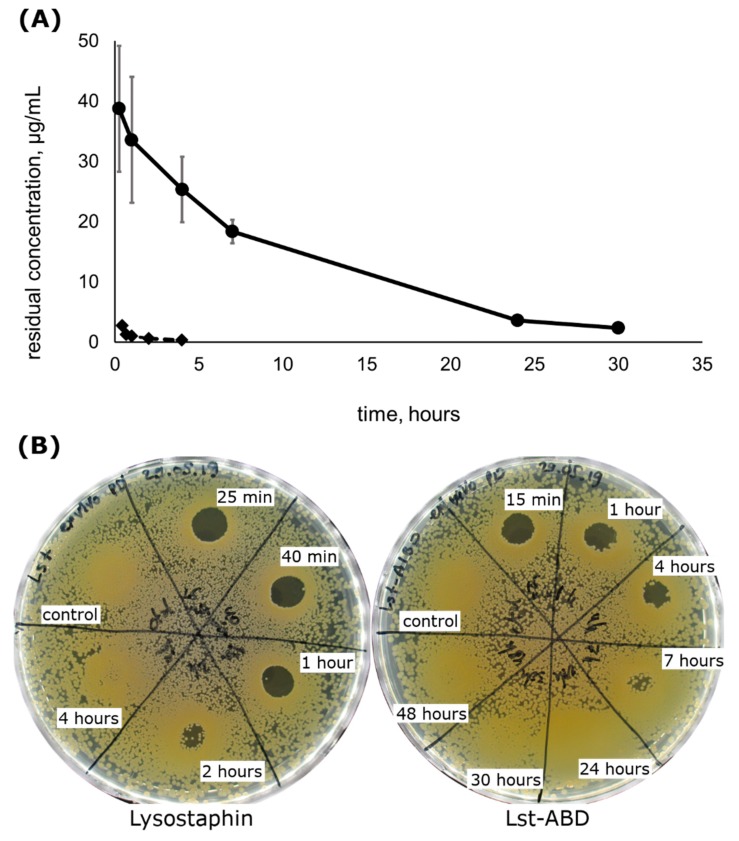
Pharmacokinetics and ex vivo pharmacodynamics of Lst-ABD in comparison to lysostaphin. (**A**) Residual concentrations of Lst-ABD (circles) and lysostaphin (diamonds) in rat plasma. Mean values from *n* = 3 (Lst-ABD) and *n* = 4 (lysostaphin) animals are shown, the error bars represent standard deviation. (**B**) Residual bactericidal activity of rat plasma towards *S. aureus* ATCC 29,213 at different timepoints after injection. Lysostaphin pharmacokinetics data in (**A**) are reproduced from [25], ex vivo pharmacodynamics data for both proteins in (**B**) were obtained in this work.

Since Lst-ABD protein retained a significant portion of the staphylolytic activity, we tested the influence of the albumin-binding domain on the rate of elimination of Lst-ABD from systemic circulation. Rats received a single intravenous injection of Lst-ABD, and the residual concentration of the protein in rat plasma was followed up to 48 h post injection (Figure 3A). Lst-ABD was cleared from rat blood significantly slower than lysostaphin, with a terminal half-life of 7.4 ± 0.5 h and the area under the curve (AUC) of 405 ± 59 mg h/L, compared to 1.5 ± 0.7 h (*p* = 4 × 10^−5^) and 3.5 ± 2.4 mg h/L (p = 0.007) of lysostaphin under the same conditions [25]. The maximal plasma concentrations C_max_ of both proteins were observed at the first sampling timepoint and constituted 38.7 ± 10.4 µg/mL for Lst-ABD and 2.8 ± 0.2 µg/mL for lysostaphin.

### 2.5. Lst-ABD Has Improved Pharmacodynamics Ex Vivo

The pharmacodynamics of lysostaphin and Lst-ABD were estimated ex vivo by testing the bactericidal activity of plasma samples obtained in the pharmacokinetics experiment. To that end, pooled plasma samples were applied onto the agar plates inoculated with *S. aureus*, and the presence of the lysis zones was documented. Plasma from the rats that received lysostaphin injection demonstrated bactericidal activity up to 2 h post injection, and no activity could be detected 4 h post injection (Figure 3B). On the contrary, plasma from the rats receiving Lst-ABD was clearly bactericidal 4 h post-injection, and some level of bactericidal activity could be observed 7 h post injection. No lysis zones were observed 24 h post injection or later (Figure 3B). Interestingly, denser bacterial growth was seen on the agar plates where the plasma samples were applied, indicating the stimulatory effect of plasma on *S. aureus* proliferation on brain heart infusion (BHI) agar (Figure 3B).

## 3. Discussion

In this work, we constructed a fusion protein consisting of an antibacterial enzyme lysostaphin and an albumin-binding domain. Although the resulting Lst-ABD protein had decreased bactericidal activity, this decrease in activity was compensated by a dramatically increased terminal half-life and AUC upon intravenous injection, as Lst-ABD demonstrated superior ex vivo pharmacodynamics.

The ability of Lst-ABD to interact with the RSA was confirmed using a photonic crystal-based biosensor. The curves of the RSA binding to and dissociation from the immobilized Lst-ABD were adequately described by a heterogeneous binding model with two independent binding sites with identical (or very similar) *k_off_* rates of 5.9 × 10^−5^ s^−1^ and ten-fold different *k_on_* rates of 2.6 × 10^4^ and 3.0 × 10^3^ M^−1^ s^−1^. Presumably, this heterogeneity stems from heterogenous immobilization of Lst-ABD on the photonic crystal chip surface. Lst-ABD was covalently immobilized by amine coupling. Since this protein possesses more than 20 lysin residues, as well as an N-terminal amine, it could be immobilized in many different orientations and conformations. Some of these orientations might be less favorable for albumin binding than the others. Alternatively, some of the molecules might be immobilized in such an orientation that requires a conformational rearrangement before binding to albumin, which would result in the decrease in *k_on_* value but would not affect *k_off_*. Regardless, the *K*_D_ of the high-affinity site was measured to be 2.3 nM and is likely to be close to the true *K*_D_ in solution. This *K_D_* value was similar to the reported *K*_D_ of the same domain in complex with human serum albumin (1.2 nM) [39]. Thus, the ability of the albumin-binding domain to interact with the rat serum albumin was largely unaffected in the Lst-ABD fusion protein.

The bacteriolytic activity of Lst-ABD was decreased compared to lysostaphin. The turbidimetric assay demonstrated a four-fold decrease in the specific activity of albumin-bound Lst-ABD compared to lysostaphin. However, Lst-ABD was still bactericidal and could eradicate most tested *S. aureus* strains in the MIC assay. This is in contrast to the PEGylated variants of lysostaphin [24] and Cpl-1 [27] that were virtually inactive. The only exception was mono-PEGylated lysostaphin, which retained a considerable portion of the bactericidal activity. However, production of the latter variant required additional purification steps, and the pharmacokinetics of mono-PEGylated lysostaphin was not reported. Very recently, several variants of an anti-staphylococcal lysin LysK fused with an albumin-binding domain were described [41]. In the presence of human serum albumin, the most potent variant was about six times less active than the parent lysin, similar to our results. Thus, fusion to an albumin-binding domain might be a generally applicable approach to the improvement of the pharmacokinetics of antibacterial lysins, since it does not result in their complete inactivation.

The pharmacokinetic parameters of Lst-ABD were dramatically improved compared to lysostaphin. The terminal half-life in rats increased almost five-fold and was 7.4 ± 0.5 h, while the AUC, which reflects the cumulative exposure of the organism to the drug, increased more than 115 times. Most of the administered lysostaphin was eliminated during the first 25 min post injection, with a slower decrease in the protein concentration during the terminal elimination phase [25]. On the contrary, the Lst-ABD clearance rate appeared to be the same over the course of the experiment. Thus, although the terminal half-life of lysostaphin is five times shorter than that of Lst-ABD, the initial fast elimination of lysostaphin is responsible for the more dramatic differences in the AUCs and C_max_ of the studied proteins. Interestingly, improvement in the pharmacokinetic parameters of lysostaphin due to the binding to albumin was much better than for the recently reported LysK–albumin-binding domain fusion C[ABD]SL [41]. The serum circulation half-life of the parent LysK variant CSL in mice was 23 h, while the same parameter for C[ABD]SL was 34 h, only 1.5 times greater. This discrepancy could probably be attributed to the unusually high serum half-life of CSL, well above that of any lysin reported so far.

Most importantly, Lst-ABD demonstrated superior ex vivo pharmacodynamics compared to lysostaphin. The plasma samples from the rats that received lysostaphin demonstrated bactericidal activity 2 h post injection but had no effect 4 h post injection. In contrast, plasma samples from the rats that received Lst-ABD were clearly bactericidal 4 h post injection, and certain bactericidal activity was present even 7 h post injection, which was 3.5 times longer compared to lysostaphin. Thus, the decrease in the staphylolytic activity of Lst-ABD is compensated by the improved pharmacokinetics, allowing it to maintain bactericidal properties in plasma for a longer time. It should be noted that the prolonged bactericidal activity of Lst-ABD in plasma might not translate to the superior in vivo pharmacodynamics. Due to its higher activity, the cumulative bacterial killing by lysostaphin might still be greater than Lst-ABD if the majority of the bacteria are killed in the first minutes after the injection of the drug. A separate experiment on an animal model of systemic staphylococcal infection is required to investigate this. On the other hand, it could be possible to improve the bacteriolytic activity of Lst-ABD by selecting the optimal linker between lysostaphin and the albumin-binding domain or choosing a different albumin-binding module. We think that optimizing the in vitro activity of a protein before extensive in vivo testing on infection models is a good practice for ethical reasons. Nonetheless, to the best of our knowledge, Lst-ABD is the first example of an antibacterial lysin with significantly prolonged bactericidal activity in the systemic circulation.

## 4. Materials and Methods

### 4.1. Cloning, Expression, and Purification

The gene coding for the Lst-ABD protein flanked by NcoI and Kpn2I restriction sites was synthesized by Evrogen (Evrogen JSC, Moscow, Russia) and cloned into pQE6 plasmid, resulting in plasmid pL475. The plasmid was transformed into *Escherichia coli* strain M15 for protein production. The nucleotide and amino acid sequences of Lst-ABD are available in the Supplementary Materials. The construction of pQE6-based plasmid pL330 coding for recombinant lysostaphin was described previously [42].

The recombinant lysostaphin was produced and purified as described before [25,42]; the same protocol was used for the production and purification of Lst-ABD. Briefly, an *E. coli* strain harboring pL475 plasmid was cultivated in LB medium in 500 mL flasks at 37 °C in the presence of antibiotics until the optical density reached 1.0. Synthesis of the recombinant protein was induced by the addition of isopropyl-β-thiogalactoside, and the culture was further incubated for 3 h at 37 °C. The cells were harvested by centrifugation and lysed with lysozyme and sonication. The obtained cell lysate was centrifuged, and the supernatant was loaded onto a WorkBeads 40S (Bio-Works technologies, Uppsala, Sweden) cation-exchange column, washed, and Lst-ABD protein was eluted with 50 mM to 500 mM NaCl gradient. The eluted protein was precipitated with 50% saturated ammonium sulfate. The precipitated pellet was dissolved in distilled water, dialyzed against distilled water, and cleared by centrifugation to remove undissolved aggregates. The obtained protein solutions were aliquoted and stored at −70 °C. The Lst-ABD yield was 16 mg per g of wet cell biomass, similar to the yield of recombinant lysostaphin [42]. The albumin-binding domain of purified Lst-ABD was found to degrade upon prolonged (more than a week) storage at +4 °C, resulting in the release of the unmodified lysostaphin. Thus, for every experiment, an aliquot was thawed and discarded after the experiment. Concentrations of the proteins were measured by a bicinchoninic acid (BCA) microplate assay (Applichem Panreac, Darmstadt/Barcelona, Germany/Spain) with BSA (Sigma-Aldrich, St. Louis, MO, USA) as standard.

The purities of the Lst-ABD and lysostaphin were assessed by SDS-PAGE. The purified Lst-ABD and lysostaphin were mixed with reducing Laemmli sample buffer for electrophoresis, heated at 95 °C for 5 min, and subjected to SDS-PAGE in 15% polyacrylamide gel. The Precision Plus Protein^TM^ Dual Color Standards (Bio-Rad Laboratories Inc, Hercules, CA, USA) were used as molecular mass markers. The gel was stained with Coomassie Brilliant Blue and documented using the GelDoc XR+ system (Bio-Rad Laboratories Inc, Hercules, CA, USA). The protein bands were quantified using Fiji software [43]. 

The correspondence of the purified Lst-ABD to the theoretical sequence was assessed by mass spectrometry analysis as described in [25]. Briefly, the protein band was excised from the gel, reduced with dithiothreitol, alkylated by iodoacetamide, and digested by trypsin. The resulting peptides were extracted by sonication in acetonitrile/formic acid/water mixtures and dried under vacuum. The peptides were reconstituted in 0.1% formic acid and analyzed using a MALDI-TOF UltrafleXtreme mass spectrometer (Bruker Daltonics, Bremen, Germany). The peaks corresponding to autolytic fragments of trypsin and keratin were used as internal standards.

### 4.2. Binding to Albumin

Interaction of Lst-ABD with rat serum albumin (RSA) was analyzed using an EVA 2.0 photonic crystal-based biosensor (www.pcbiosensors.com). The principle of this method is conceptually similar to the surface plasmon resonance (SPR)-based approach. Briefly, the polarized laser beam is directed through an optical system onto the one-dimensional photonic crystal (1D PC) consisting of several alternating layers of SiO_2_ and Ta_2_O_5_, and the reflected light is detected by a CMOS chip. The opposite side of the PC faces the flow cell and is functionalized with the ligand that interacts with the analyte pumped through the flow cell. The use of the photonic crystal allows to simultaneously monitor both the change in the adsorbed layer thickness and the refractive index of the medium in the flow cell due to the existence of *s*-polarized and *p*-polarized surface waves. Thus, the sensogram can be automatically corrected for the change in the medium refractive index [40].

To clean the PC chip surface, the chips were sonicated for 5 min in acetone and for 5 min in ethanol (S-15 Elmasonic ultrasonic cleaning unit, Elma Schmidbauer, Singen, Germany), air-dried, treated with UV for 10 min twice with 5 min sonication in ethanol between UV treatments, again sonicated in acetone and ethanol for 5 min each, and dried at 55 °C for 20 min. To derivatize the chip surface with carboxylate groups, the chips were treated for 3–4 h with 1% (3-triethoxysilyl)propylsuccinic anhydride (TESPSA) (Gelest, Morrisville, PA, USA) and 0.5% *N*,*N*-diisopropylethylamine (DIPEA, Sigma-Aldrich, St. Louis, MO, USA) in anhydrous toluene. After that, the chips were washed twice with acetone, ethanol, and distilled water and dried at 100–110 °C for 30 min.

Carboxylate-derivatized chips were assembled with the single-channel flow cell (cell volume 5–7 µL) and mounted on the EVA 2.0 instrument according to manufacturer’s instructions. The chip surface was rinsed with sodium phosphate buffer (0.1 M Na_2_HPO_4_, pH 6.2) for 5 min, and Lst-ABD was covalently immobilized on the chip surface in situ by standard amine coupling using 1-ethyl-3-(3-dimethylaminopropyl)carbodiimide/*N*-hydroxysuccinimide (EDC/NHS) chemistry. To that end, the chip surface was activated by running EDC/NHS (50 mg/mL each) water solution through the flow cell for ~20 min at 0.07 mL/min flow rate, washing the unreacted EDC/NHS with MES buffer (50 mM, pH 5.0) for 3 min, and running 40 µg/mL Lst-ABD in MES buffer until the sensogram reached the plateau (~10 min). The surface was deactivated with 1 M ethanolamine (~5 min), flushed with 0.05M HCl/10 mM glycine solution for 5 min, and washed with PBST buffer (PBS + 0.02% Tween-20, pH 7.4) until stabilization of the baseline. Different concentrations of RSA in PBST were then pumped through the flow cell to analyze association and dissociation kinetics of the Lst-ABD/RSA complex in the following cycle: ~5 min association phase with RSA in PBST, ~5 min dissociation phase with PBST without RSA, regeneration of the surface by glycine/HCl solution for 30 s, and baseline stabilization with PBST.

The results were analyzed using a simple kinetic model for heterogeneous binding with two independent binding sites. First, single *k_off_* was determined by fitting into the dissociation phase of the kinetic curves (Equation (1)). Then, *k*_*off*1_ = *k*_*off*2_ = *k_off_* were fixed, and *k*_*on*1_, *k*_*on*2_, *R*_*max*1_, and *R*_*max*2_ were determined by fitting into the association phase of the kinetic curves (Equation (2)). Attempts to fit all the parameters simultaneously using both association and dissociation phases resulted in very similar *k*_*off*1_ and *k*_*off*2_ values that, however, differed significantly between the experiment replications. Thus, we had to use the stepwise fitting procedure and set *k*_*off*1_ = *k*_*off*2_.

(1) $$\frac{dR_{t}}{dt}=-k_{off}\cdot R_{t}$$

(2) $$\frac{dR_{t}}{dt}=k_{on1}\cdot C\cdot \left(R_{max1}-R_{t1}\right)-k_{off}\cdot R_{t1}+k_{on2}\cdot C\cdot \left(R_{max2}-R_{t2}\right)-k_{off}\cdot R_{t2}$$

The code used for data analysis is available as a Jupyter notebook in the Supplementary Materials, along with the raw data from the EVA 2.0 device.

### 4.3. Antibacterial Activity

Antibacterial activity of Lst-ABD was assessed as previously described [25], with minor modifications to include RSA in certain experimental variants.

The minimum inhibitory (MIC) and minimum bactericidal (MBC) concentrations of Lst-ABD and lysostaphin were measured by a microplate dilution assay in Mueller–Hinton broth (Sifin Diagnostics, Berlin, Germany) with 2% NaCl and 0.1% BSA. *S. aureus* strain ATCC 29,213 and 11 *S. aureus* clinical isolates from the local collections of N.F. Gamaleya Research Center (Moscow, Russia) were used in this work. *S. aureus* strains were cultivated overnight on BHI agar (Sifin Diagnostics, Berlin, Germany) plates and suspended in saline to an optical density of 0.5 McFarland. The suspensions were diluted 300 times in assay medium to an approximate bacterial concentration of 5 × 10^5^ CFU/mL, and the resulting suspensions were pipetted into 96-well plates (90 µL per well) (Costar 3599, Corning, Corning, NY, USA). Then, 10 µL of two-fold serial dilutions of Lst-ABD or lysostaphin in the same medium were added. The plates were incubated in a plate shaker (PST-60 HL plus, Biosan, Riga, Latvia) at 37 °C and 400 rpm for ~20 h. The MIC was determined as the lowest concentration of the protein that prevented visible bacterial growth. After determination of the MIC, 3 µL of the well contents were transferred to a BHI agar plate and incubated overnight at 37 °C. The minimum concentration of the protein that prevented bacterial growth on the agar plate was assumed to be the minimum bactericidal concentration (MBC). To test the influence of albumin on the Lst-ABD MIC and MBC, serial dilutions of Lst-ABD were prepared in the presence of 0.5 mg/mL RSA so that the final concentration of RSA in the assay mixture was 50 µg/mL. The sensitivity of the tested *S. aureus* strains to methicillin was checked by disk diffusion assay on Mueller–Hinton agar (Sifin Diagnostics, Berlin, Germany) using 30 µg cefoxitin disks (HiMedia Laboratories Pvt. Limited, Mumbai, India).

The kinetics of staphylococcal cell lysis by Lst-ABD was assessed by following the reduction in the turbidity of bacterial cell suspension in the presence of Lst-ABD as described [25]. Briefly, *S. aureus* ATCC 29,213 was cultivated on a BHI agar plate, inoculated in Mueller–Hinton broth with 2% NaCl, and incubated overnight at 37 °C and 100 rpm. Bacterial cells were harvested by centrifugation, washed with Tris buffered saline (TBS, 50 mM Tris, 150 mM NaCl, pH 7.5), and resuspended in TBS with 0.1% BSA at the optical density of 4.0 McFarland. This bacterial suspension was accurately pipetted into the wells of a 96-well plate (180 µL per well) to avoid bubble formation, the plate was incubated at 37 °C and 400 rpm for 10 min, and 20 µL of different concentrations of Lst-ABD with or without 1 mg/mL RSA were added to the wells (final concentration of RSA 100 µg/mL). The plate was incubated at 37 °C and slow shaking in a Multiscan FC microplate reader (Thermo Fischer Scientific, Waltham, MA, USA), and the optical density of the suspension was measured at 550 nm every minute during a period of 1 h. Within one experiment, the measurements were done in triplicates for every Lst-ABD concentration, and the experiment was performed three times. For every protein concentration, the rate of turbidity reduction was determined as the slope of the linear portion of the turbidity curve. Then, the dependence on the turbidity reduction rate on the protein concentration was approximated by a linear function and expressed as ∆OD/min µM protein [44].

### 4.4. Pharmacokinetics

The pharmacokinetic parameters of Lst-ABD were determined as previously described for lysostaphin [25]. Female Wistar rats (*n* = 3) weighing ~300 g received bolus intravenous injections of 0.5 mg Lst-ABD in 500 µL of saline in the tail vein. The blood was sampled at 15 min and at 1, 4, 7, 24, 30, and 48 h post injection, and the plasma was separated from blood and stored at −80 °C. The residual concentration of Lst-ABD in rat plasma was determined using sandwich ELISA. Briefly, 96-well plates (Costar 3590, Corning, Corning, NY, USA) were coated with rabbit polyclonal anti-lysostaphin serum in carbonate–bicarbonate buffer overnight at +4 °C, and washed and blocked with BSA for 3 h at 37 °C. After that, plasma samples diluted 1/2000 in TBS were added, and the plate was incubated overnight at +4 °C. Then, the plate was washed, murine monoclonal antibodies 2F9 (State Research Center for Applied Microbiology and Biotechnology, Obolensk, Russia) in TBS with 0.05% Tween 20 and 1% BSA were added, and the plate was again incubated overnight at +4 °C. After incubation with detection antibodies, the plate was washed, filled with the solution of HRP-conjugated secondary anti-mouse antibodies (IMTEK, Moscow, Russia), incubated for 3 h at 37 °C, and washed and filled with TMB peroxidase substrate for 25 min. The reaction was stopped with sulfuric acid, and the optical density was measured at a wavelength of 450 nm.

The terminal half-life and area under the curve were calculated following the noncompartmental approach [45]. When plotted in log-linear coordinates, the Lst-ABD residual concentration in plasma demonstrated a linear decrease with time; thus, all timepoints were used to calculate the terminal half-life. The AUC was calculated by the trapezoid rule starting from the first timepoint and using the terminal half-life to extrapolate the concentration to zero level.

The experiment was approved by N.F. Gamaleya Research Center biomedical ethics committee, protocol №15 16.10.2018.

### 4.5. Ex Vivo Pharmacodynamics

The protocol for the estimation of ex vivo bactericidal activity of plasma samples was adapted from Ref. [6]. Plasma samples from all rats corresponding to the same group and the same time point were pooled. Plasma samples from rats receiving lysostaphin were obtained previously [23], and plasma samples from rats receiving Lst-ABD were obtained in the present work. Fresh overnight culture of *S. aureus* ATCC 29,213 on BHI agar plates was resuspended in saline to the optical density of 0.5 McFarland and diluted 100 times to ~1.5 × 10^6^ CFU/mL. The resulting suspension was spread over BHI agar plates (300 µL per plate, 3 plates per protein variant) and allowed to absorb for 10 min. After that, 10 µL drops of pooled plasma samples were spotted onto the agar plates and allowed to absorb for 20 min. The plates were incubated at 37 °C for 24 h, and the presence of the lysis zones was documented.

### 4.6. Statistical Analysis

All results are presented as mean ± standard deviation. Statistical analysis was performed using Real Statistics Resource Pack add-on for Microsoft Excel (Release 4.7, copyright (2013–2017) Charles Zaiontz, www.real-statistics.com). The differences in bacteriolytic activity were analyzed by one-way ANOVA with Tukey’s Honest Significant Difference post hoc analysis. The differences between terminal half-lives and AUC were compared by two-tail Student’s *t*-tests with unequal variances.

## Figures and Tables

**Table 1:** Parameters of RSA interaction with immobilized Lst-ABD

Parameter	Mean ± Stdev
*k_off_*	5.9 × 10^−5^ ± 2.0 × 10^−5^ s^−1^
*k*_*on*1_	2.6 × 10^4^ ± 8.4 × 10^2^ M^−1^ s^−1^
*k*_*on*2_	3.0 × 10^3^ ± 4.3 × 10^1^ M^−1^ s^−1^
*K*_D1_	2.3 ± 0.8 nM
*K*_D2_	20.0 ± 6.6 nM

**Table 2:** Minimum inhibitory concentration (MIC) and minimum bactericidal concentration (MBC) values of lysostaphin, Lst-ABD, and Lst-ABD in the presence of RSA against different *Staphylococcus aureus* isolates.

Strain	MSSA/MRSA	MIC, µg/mL			MBC, µg/mL		
		Lysostaphin	Lst-ABD	Lst-ABD + RSA	Lysostaphin	Lst-ABD	Lst-ABD + RSA
ATCC 29213	MSSA	0.1^1^	0.8	1.6	0.1^1^	0.8	1.6
Z 715-18	MSSA	0.4	>12.8	>12.8	0.4	>12.8	>12.8
Z 76-19	MSSA	0.05	3.2	3.2	0.05	3.2	3.2
Z 88-19	MSSA	0.05	0.8	1.6	0.05	0.8	1.6
F 832-14	MRSA	0.1	3.2	3.2	0.1	3.2	3.2
R 81-19	MSSA	0.05	3.2	3.2	0.05	3.2	3.2
In 0102-19	MSSA	0.025	1.6	1.6	0.05	1.6	3.2
R 116-14	MRSA	0.05	1.6	3.2	0.05	3.2	3.2
Z 73-14	MSSA	0.05	3.2	6.4	0.1	3.2	6.4
301	MRSA	0.05	1.6	1.6	0.05	1.6	1.6
629	MRSA	0.05	1.6	3.2	0.05	1.6	3.2
247G	MRSA	0.4	12.8	12.8	0.4	12.8	12.8

^1^ from [23].

## Supplementary Materials

The following are available online: nucleotide and amino acid sequences of Lst-ABD; code used to for the albumin binding data analysis and raw data from EVA 2.0 biosensor.
