# Supplementary material for: Fusion of Lysostaphin to an Albumin Binding Domain Prolongs Its Half-Life and Bactericidal Activity in the Systemic Circulation
Source: Molecules. 2019 Aug 9;24(16):2892. doi: 10.3390/molecules24162892 (PMC6719061; doi:10.3390/molecules24162892)
Supplement: Supplementary file 1 [file molecules-24-02892-s001.zip › Supplementary/binding_to_albumin.zip!/binding_to_albumin/binding_to_albumin.html]

binding\_to\_albumin


## Binding of Lst-AlBD to rat serum albumin¶

The interaction between Lst-AlBD and rat serum albumin (RSA) was analysed using EVA 2.0 photonic crystal biosensor. the principle of EVA 2.0 biosensor is analogous to the surface plasmon resonance biosensors. One interaction partner (ligand) is immobilized on the surface of the biosensor, and the other one (analyte) is pumped through the flow cell, so that the constant concentratio nof the analyte in the solution is maintained. The analyte binds the immobilized ligand and this binding is detected by the biosensor, resulting in the association curve. To obtain the dissociation curve, the buffer solution is pumped through the flow cell and the process of spontaneous detachment of the bound analyte is recorded. The association and dissociation curves recorded at the different concentrations of the analyte can be used to fit the kinetic parameters (Kon, Koff), which in turn are used to calculate the affinity of the complex (Kd).

In this experiment we used immobilized Lst-AlBD as ligand and RSA as analyte. The reverse orientation was not possible due to the non-specific interaction of Lst-AlBD with the surface of the biosensor. RSA was pumped through the flow cell at the concentrations of 1.5 mg/mL, 250 ug/mL, 42 ug/mL, 7 ug/mL and 1.2 ug/mL. The experiment was performed on two separate days, and on the second day the full set of concentrations was run twice. Thus, three datasets for the full set of RSA concentrations (one on the first day and two on the second day) were obtained, analyzed, and used to calculate the average affinity constants.

The code below was used to analyze the data, calculate the affinity constants and generate the figures.

## Data loading and preparation¶

Import the auxiliary functions to read and manipulate data from the EVA 2.0 biosensor

In [1]:

```
import numpy
import eva
```

Load the data for an overview. The raw data from the EVA 2.0 device are stored in the daResults.dat file.

In [2]:

```
raw_data = []
for filename in ("data\\daResults_190918.dat", "data\\daResults_200918.dat"):
    raw_data.append(eva.read_daResults(open(filename)))
```

In [3]:

```
%matplotlib inline
import matplotlib.pyplot as pyplot

# the EVA 2.0 device registeres the binding of the analyte in several "channels" across the biosensor surface,
# in this case 12 channels were used.
num_channels = 12

for d in raw_data:
    time = d[:,1] # the second column in the data file is the time in seconds
    channels = [d[:,x] for x in range(4, 4+num_channels)] # the binding data starts at column 5
    for channel in channels:
        pyplot.plot(time, channel)
    pyplot.show()
```

These figures can be used to assess the quality of the sensograms. For example, in the first dataset there is erroneous signal in one of the channels near the end of the experiment. The data from this channel will have to be omitted.
These figures are also used to determine the time-points of the beginning and the end of every injection. These time-points are manually extracted from the sensograms and stored in a injections.csv file, along with the concentrations of the reagents and other relevant information.

Read in the injections.csv files manually prepared after the previous step and split the raw data into collections of injections.

In [4]:

```
injections_190918 = eva.parse_injections_file(open("data\\injections_190918.csv"), raw_data[0])
injections_200918 = eva.parse_injections_file(open("data\\injections_200918.csv"), raw_data[1])

# remove the erroneous channel in the first experiment
for inj in injections_190918:
    inj.channel_mask[0] = 0
    inj.apply_channel_mask()

# adjust the data to start from zero time and zero signal level
for inj in injections_190918 + injections_200918:
    inj.zero_align()

# sort the data into sets of injections
# there was one set of injections of different concentrations of albumin in the first experiment
# and two sets of injections in the second experiment
data = []
data.append(injections_190918[1:5]) # the highest concentration was omitted as it was not well reproduced
data.append(injections_200918[1:5]) 
data.append(injections_200918[6:])
```

Plot the sensograms for visual inspection.

In [5]:

```
for i in range(3):
    dataset = data[i]
    color = ['r', 'g', 'b'][i]
    for inj in dataset:
        pyplot.plot(inj.zero_timeline, inj.zero_mean_signal, color)
pyplot.show()
```

## Data analysis¶

The kinetic models for association and dissociation phases.

In [6]:

```
from scipy.integrate import odeint

# t - time
# Rmax - maximal signal level when all the binding sites are occupied
# Kon, Koff - kinetic constants
# C - analyte concentration
# Rstart - the signal level at the beginning of the dissociation phase

def association_de(t, Rmax, Kon, Koff, C):
    y0 = 0
    de = lambda y, t: Kon*C*Rmax - Kon*C*y - Koff*y
    return odeint(de, y0, t).flatten()

def dissociation_de(t, Koff, Rstart):
    y0 = Rstart
    de = lambda y, t: -Koff*y
    return odeint(de, y0, t).flatten()
```

Simple one-site model could not be fitted well into the obtained experimental curves. Thus, we used heterogenous model with two independent binding sites. However, when both association and dissociation phases were simultaneously used for fitting the parameters, the Koff values were poorly reproduced between the experiments. Thus, we first fitted a single Koff value to the dissociation phase, and then used this Koff value to fit two independent Kon values to the association phase. This procedure resulted in a good fit and a good agreement between the experiments.

Fit the Koff kinetic constant into the datasets.

In [7]:

```
from scipy.optimize import minimize

def min_dissociation(Koff, injections):
    Koff = numpy.power(10, Koff) # transform the constant back from the log-transformed value used in minimization
    result = 0
    for inj in injections:
        t_dis = inj.dis_timeline
        y_dis = inj.dissociation
        Rstart = inj.association[-1]
        dis = dissociation_de(t_dis, Koff, Rstart)
        result += numpy.power(y_dis-dis, 2).sum()
    return result

Koffs = []

for i, injections in enumerate(data):
    result = minimize(min_dissociation, x0=[-4], args=(injections), method='Nelder-Mead', options={'maxfev': 10000})
    Koff = result.x[0]
    Koffs.append(Koff)
    print "\nResult %i:" % (i+1)
    print result.message
    print "Koff = %3.2E" % numpy.power(10, Koff)
```

```
Result 1:
Optimization terminated successfully.
Koff = 3.30E-05

Result 2:
Optimization terminated successfully.
Koff = 6.25E-05

Result 3:
Optimization terminated successfully.
Koff = 8.15E-05
```

Fit the Rmax values and Kon kinetic constants into the datasets using the two-site heterogenous binding model and Koff constants obtained in the previous step.

In [8]:

```
def min_association(opt_params, injections, Koff):
    Rmax1, Kon1, Rmax2, Kon2 = opt_params
    # back from log-transformed values
    Kon1, Kon2 = numpy.power(10, [Kon1, Kon2])
    result = 0
    for inj in injections:
        t_ass = inj.ass_timeline
        y_ass = inj.association
        C = inj.analyte_concentration
        ass1 = association_de(t_ass, Rmax1, Kon1, Koff, C)
        ass2 = association_de(t_ass, Rmax2, Kon2, Koff, C)
        ass = ass1 + ass2
        result += numpy.power(y_ass-ass, 2).sum()
    return result

Rmaxs1, Kons1, Rmaxs2, Kons2 = [], [], [], []

for i in range(3):
    injections = data[i]
    Koff = numpy.power(10, Koffs[i])
    res = minimize(min_association, x0=[1.5, 4, 1.5, 3], args=(injections, Koff), method='Nelder-Mead', options={'maxfev': 10000})
    Rmaxs1.append(res.x[0])
    Kons1.append(res.x[1])
    Rmaxs2.append(res.x[2])
    Kons2.append(res.x[3])
    print "\nResult %i:" % (i+1)
    print result.message
    print "Rmax1 = %3.2f\nKon1 = %3.2E\nRmax2 = %3.2f\nKon2 = %3.2E" % (res.x[0], numpy.power(10, res.x[1]), res.x[2], numpy.power(10, res.x[3]))
```

```
Result 1:
Optimization terminated successfully.
Rmax1 = 1.21
Kon1 = 2.48E+04
Rmax2 = 1.39
Kon2 = 2.99E+03

Result 2:
Optimization terminated successfully.
Rmax1 = 1.14
Kon1 = 2.67E+04
Rmax2 = 1.33
Kon2 = 2.93E+03

Result 3:
Optimization terminated successfully.
Rmax1 = 1.17
Kon1 = 2.52E+04
Rmax2 = 1.29
Kon2 = 3.03E+03
```

Plot the theoretical and experimental curves for visual inspection.

In [9]:

```
for i in range(3):
    injections = data[i]
    for inj in injections:
        t_ass = inj.ass_timeline
        y_ass = inj.association
        t_dis = inj.dis_timeline
        y_dis = inj.dissociation
        C = inj.analyte_concentration
        Rmax1, Rmax2 = Rmaxs1[i], Rmaxs2[i]
        Kon1, Kon2, Koff = numpy.power(10, (Kons1[i], Kons2[i], Koffs[i]))
        # association
        ass1 = association_de(t_ass, Rmax1, Kon1, Koff, C)
        ass2 = association_de(t_ass, Rmax2, Kon2, Koff, C)
        ass = ass1 + ass2
        # dissociation
        Rstart = ass[-1] # the last point of the theoretical association curve
        dis = dissociation_de(t_dis, Koff, Rstart)
        # plot!
        pyplot.plot(t_ass, y_ass, 'b')
        pyplot.plot(t_ass, ass, 'g')
        pyplot.plot(t_dis+t_ass[-1], y_dis, 'b')
        pyplot.plot(t_dis+t_ass[-1], dis, 'g')
    pyplot.show()
```

Calculate the average values for the kinetic and equilibrium constants.

In [10]:

```
Koffs, Kons1, Kons2 = numpy.power(10, Koffs), numpy.power(10, Kons1), numpy.power(10, Kons2)
```

In [11]:

```
mean_Koff, std_Koff = numpy.mean(Koffs), numpy.std(Koffs)
mean_Kon1, std_Kon1 = numpy.mean(Kons1), numpy.std(Kons1)
mean_Kon2, std_Kon2 = numpy.mean(Kons2), numpy.std(Kons2)
Kds1 = numpy.array(Koffs) / numpy.array(Kons1)
Kds2 = numpy.array(Koffs) / numpy.array(Kons2)
mean_Kd1, std_Kd1 = numpy.mean(Kds1), numpy.std(Kds1)
mean_Kd2, std_Kd2 = numpy.mean(Kds2), numpy.std(Kds2)

print "Koff = %3.1E +/- %3.1E" % (mean_Koff, std_Koff)
print "Kon1 = %3.1E +/- %3.1E" % (mean_Kon1, std_Kon1)
print "Kon2 = %3.1E +/- %3.1E" % (mean_Kon2, std_Kon2)
print "Kd1 = %3.1E +/- %3.1E" % (mean_Kd1, std_Kd1)
print "Kd2 = %3.1E +/- %3.1E" % (mean_Kd2, std_Kd2)
```

```
Koff = 5.9E-05 +/- 2.0E-05
Kon1 = 2.6E+04 +/- 8.4E+02
Kon2 = 3.0E+03 +/- 4.3E+01
Kd1 = 2.3E-09 +/- 7.8E-10
Kd2 = 2.0E-08 +/- 6.6E-09
```

Generate a picture for the manuscript.

In [27]:

```
fig, axes = pyplot.subplots(figsize=(4.2,2.5), dpi=300)

# plot the experimental and theoretical curves
injections = data[1] # choose the second set of injections
for inj in injections:
    t_ass = inj.ass_timeline
    y_ass = inj.association
    # cut off the plot at 500 sec so that the picture looks neat :)
    end_point = eva._get_nearest_number(inj.dis_timeline, 500 - inj.ass_timeline[-1])[1]
    t_dis = inj.dis_timeline[:end_point]
    y_dis = inj.dissociation[:end_point]
    C = inj.analyte_concentration
    Rmax1, Rmax2, Kon1, Kon2, Koff = Rmaxs1[1], Rmaxs2[1], Kons1[1], Kons2[1], Koffs[1]
    # association
    ass1 = association_de(t_ass, Rmax1, Kon1, Koff, C)
    ass2 = association_de(t_ass, Rmax2, Kon2, Koff, C)
    ass = ass1 + ass2
    # dissociation
    Rstart = ass[-1] # the last point of the theoretical association curve
    dis = dissociation_de(t_dis, Koff, Rstart)
    # plot!
    pyplot.plot(t_ass, y_ass, 'k')
    pyplot.plot(t_ass, ass, 'r')
    pyplot.plot(t_dis+t_ass[-1], y_dis, 'k')
    pyplot.plot(t_dis+t_ass[-1], dis, 'r')

# plot the BSA injection for comparison
BSA_inj = injections_190918[-1]
end_point = eva._get_nearest_number(BSA_inj.dis_timeline, 500 - BSA_inj.ass_timeline[-1])[1]
pyplot.plot(BSA_inj.ass_timeline, BSA_inj.association, 'b')
pyplot.plot(BSA_inj.dis_timeline[:end_point] + BSA_inj.ass_timeline[-1], BSA_inj.dissociation[:end_point], 'b')

# add vertical line between association and dissociation phases
axes.axvline(x=265, color='k', linestyle="--")

# figure style
axes.set_xlabel("time, sec")
axes.set_ylabel("thickness, nm")
pyplot.tight_layout(pad=0.5)

# save the figure
pyplot.savefig("binding_to_albumin.png", format='png', dpi=300)
```
